# Quorum-Quenching AHL-Lactonase Est816 Inhibits Polymicrobial Subgingival-Plaque-Derived Biofilm Formation

**DOI:** 10.3390/dj13080372

**Published:** 2025-08-15

**Authors:** Zelda Ziyi Zhao, Wenwen Shan, Xiaoyu Sun, Tianfan Cheng, Jing Zhang, Chun Hung Chu

**Affiliations:** 1 Faculty of Dentistry, The University of Hong Kong, Hong Kong SAR, China; zelda96@connect.hku.hk (Z.Z.Z.); chengtfc@hku.hk (T.C.); 2 Key Laboratory of Oral Diseases Research of Anhui Province, College & Hospital of Stomatology, Anhui Medical University, Hefei 230032, China; s1094556676@163.com (W.S.); 2011520001@ahmu.edu.cn (X.S.)

**Keywords:** biofilms, quorum sensing, *N*-acyl-homoserine lactones, oral microbiome, virulence factors

## Abstract

**Objectives**: This study aimed to investigate the effects of the quorum-quenching enzyme *N*-acyl-homoserine lactone (AHL)-lactonase Est816 on biofilm formation in subgingival plaque microbiota from participants with advanced periodontitis. **Methods**: Subgingival plaque samples were collected from 30 adults with untreated Stage III or higher periodontitis and cultured anaerobically. Est816 was applied in vitro, with phosphate-buffered saline (PBS) serving as the control. Biofilm composition was analyzed via 16S rRNA sequencing, and alpha diversity metrics were assessed. Differential taxa abundance was assessed with the multivariate statistical software MaAsLin3. Biofilm morphology, biomass, and thickness were evaluated using scanning electron microscopy (SEM), crystal violet staining, and confocal laser scanning microscopy (CLSM). **Results**: Est816 significantly reduced microbial richness (Chao1 Index, *p* = 0.031), biofilm biomass (64% reduction, *p* < 0.001), and thickness (76% reduction, *p* < 0.001) compared to controls. SEM revealed fragmented biofilm architecture in Est816-treated samples. **Conclusions**: AHL-lactonase Est816 inhibited polymicrobial subgingival-plaque-derived biofilm formation while reducing species richness, phylogenetic diversity, and community evenness. These findings demonstrate Est816’s potential as an adjunctive therapy for disrupting pathogenic biofilms in periodontitis.

## 1. Introduction

Periodontitis, a chronic inflammatory disease affecting over 1.1 billion people globally, is the sixth most prevalent human disease and a leading cause of tooth loss, with profound implications for oral and systemic health [1]. This multifactorial disease arises from complex interactions between subgingival microbial communities, host immune–inflammatory responses, and environmental risk factors [2,3,4,5]. The structurally organized polymicrobial biofilms serve as ecological niches for microbial persistence. These biofilms function as reservoirs for diverse virulence determinants, including microbial proteases, lipopolysaccharides, and other molecular patterns. Through these factors, the biofilm communities evade host defense mechanisms and develop resistance to conventional therapeutic interventions [6,7]. Clinical observations indicate disease recurrence rates of up to 50%, which may reflect both the intrinsic resilience of biofilms and site-specific challenges in therapy delivery (e.g., pocket anatomy and patient compliance) [8,9]. This underscores the need for novel strategies targeting biofilm persistence mechanisms.

Biofilm resilience is critically regulated by quorum-sensing (QS) systems. This mechanism coordinates biofilm maturation, virulence factor secretion, and metabolic cooperation in a density-dependent manner. In periodontal biofilms, QS relies on diffusible signaling molecules such as *N*-acyl-homoserine lactones (AHLs), which are produced by Gram-negative pathogens [10]. Critically, AHLs detected in human subgingival plaque correlate with periodontal disease severity, implicating QS as a potential therapeutic target [11]. Conventional therapies (e.g., scaling, root planning, and antibiotics) aim to reduce biofilm biomass or eradicate pathogens but lack efficacy against the QS system. This oversight allows residual bacteria to utilize QS-mediated cooperative behaviors, facilitating biofilm re-establishment and subsequent disease recurrence. The enzymatic disruption of QS signaling has emerged as a precision strategy to combat biofilm-associated infections. It offers an alternative by disrupting bacterial communication without directly killing microbes, thereby minimizing selective pressure for antibiotic resistance. While studies have explored quorum-quenching enzymes such as Aii20j (from *Tenacibaculum* sp.) and SsoPox (from *Sulfolobus solfataricus*) in oral models, their focus has been limited to early-stage biofilms or single-species systems [12,13,14,15]. Additionally, their clinical applicability remains limited by instability in the proteolytic environment, fluctuating the pH conditions of the subgingival environment.

Periodontitis progresses from gingivitis (Stage I) through increasingly severe dysbiotic stages (Stages II/III), ultimately reaching irreversible tissue destruction (Stage IV), with inflammation and microbial shifts driving disease progression through a self-perpetuating cycle that transitions from reversible commensal overgrowth to anaerobic pathogen dominance and diversity collapse. Recent hypotheses suggest the potential mechanisms for the etiopathogenesis of this condition; however, these remain to be validated by further evidence-based studies [9,16]. The primary therapeutic goal for advanced periodontitis (Stages III/IV) is the elimination of periodontal pockets. While gingivectomy achieves pocket reduction and microbiota clearance, its main drawback, less favorable aesthetic outcomes, remains a critical consideration in clinical decision making, particularly in anterior regions. Meanwhile, treatment is particularly challenging due to structural biofilm barriers and microbial interactions and disease-altered tissue microenvironments including increased vascularity and gingival crevicular fluid flow, which collectively limit therapeutic agent penetration and efficacy [17,18,19,20,21]. Despite these clinically significant barriers, quorum-quenching strategies remain underexplored in clinically derived, polymicrobial biofilms from Stage III/IV periodontitis—a critical knowledge gap given the persistent therapeutic challenges in these advanced cases.

We cloned AHL-lactonase Est816, which has a high degradation efficiency against all types of AHLs with unparalleled stability under pH-variable conditions, precisely the properties required to overcome the subgingival environment’s challenges [22,23]. This study employed an in vitro model to investigate how the quorum-quenching enzyme Est816 modulates biofilm formation in microbial communities derived from deep periodontal pockets (≥6 mm) of Stage III/IV periodontitis patients, as classified by the 2017 World Workshop criteria. This model examines how quorum quenching affects cultivable subgingival communities under controlled conditions. We hypothesize that Est816 may disrupt QS-mediated interactions in these polymicrobial subgingival-plaque-derived biofilms, potentially reducing their structural organization.

## 2. Materials and Methods

### 2.1. Cloning and Purification of Est816

The AHL-lactonase Est816 was initially identified from a metagenomic library constructed using soil samples collected from the Turban Basin region. Through high-throughput functional screening of approximately 26,000 recombinant clones (average insert size 4.5 kilobases), we isolated a positive clone designated pUC118A harboring an 816-base pair open reading frame. Bioinformatics analysis revealed that this sequence encodes a 29.9 kilodalton protein (GenBank accession JQ996501) exhibiting characteristic features of Family V esterases, including the conserved catalytic triad arrangement (Ser93-Asp214-His242) [24]. For heterologous expression, the coding sequence was PCR-amplified and directionally cloned into the pET-28a(+) expression vector, generating a C-terminally 6×His-tagged fusion construct. Recombinant protein production was achieved in E. coli BL21(DE3) cells through induction with 0.5 mM isopropyl β-D-1-thiogalactopyranoside (IPTG) at 25 °C for 10 h. The resulting 31.2 kDa fusion protein (including the affinity tag) was purified to homogeneity (>95% purity as verified by SDS-PAGE) using immobilized metal affinity chromatography with nickel-nitrilotriacetic acid (Ni-NTA) resin, with typical yields of 0.3 mg mL^−1^ purified enzyme. Catalytic activity was validated using *N*-octanoyl-L-homoserine lactone (C8-HSL) as the substrate in standard degradation assays, as described previously.

### 2.2. Subject Recruitment

This study was conducted with approval from the local Independent Review Board. Subgingival plaque samples were collected from adults diagnosed with Stage III/IV periodontitis, as defined by the American Academy of Periodontology and the European Federation of Periodontology Classification of Periodontal and Peri-implant Diseases 2017: (1) interdental clinical attachment loss of at least 5 mm and at least 2 non-adjacent teeth; (2) radiographic bone loss extending to the mid-third of the root or beyond; (3) tooth loss due to periodontitis; (4) complexity of management (e.g., furcation involvement and masticatory dysfunction) (Stage IV only). All participants provided written informed consent prior to sample collection.

Eligible participants were required to have at least 20 natural teeth. They should have radiographic evidence of alveolar bone loss and periodontal pockets with at least 6 mm at sampling sites. Exclusion criteria included periodontal treatment within 1 year, antibiotic or anti-inflammatory drug use within the past 6 months, systemic diseases including diabetes, cardiovascular disorders, immune deficiency, pregnancy, or lactation, and severe oral conditions such as alveolar abscesses.

### 2.3. Sample Collection

Subgingival plaque was collected using sterile Gracey curettes from the deepest periodontal pocket (at least 6 mm) of molar or premolar teeth per participant. Samples were pooled into 2 mL of brain–heart infusion (BHI) broth supplemented with 5 μg mL^−1^ hemin and 1 μg mL^−1^ vitamin K_1_ to support anaerobic growth. Samples were transported to the laboratory in anaerobic jars (AnaeroPacks, Mitsubishi Gas Chemical Company, Tokyo, Japan) in a Styrofoam box to maintain anaerobic conditions. Additionally, the subgingival biofilm samples were collected individually from periodontal pockets of each patient (n = 30) during their initial clinical visits and immediately processed for in vitro cultivation to maintain microbial viability. Each sample was cultured and analyzed separately to preserve patient-specific microbial profiles. Samples were collected from the deepest periodontal pocket per participant to obtain microbial communities. Such samples did not exclusively contain putative periodontal pathogens nor fully reflect disease etiology; this sampling strategy targeted ecological niches where microbial communities were typically established in periodontitis patients.

### 2.4. Biofilm Cultivation

Samples were incubated at 37 °C for 12 h in an anaerobic chamber. The 12 h pre-incubation allowed the recovery of bacteria from sampling stress before biofilm formation [12]. Bacterial suspensions were centrifuged to pellet the bacterial cells. The suspensions were normalized to an optical density (OD_600_) of 0.2 using pre-reduced BHI broth. For biofilm formation, sterilized glass coverslips were placed in 24-well plates, and each well was inoculated with 0.5 mL of bacterial suspension. Est816 was added to treatment wells at a final concentration of 6 U mL^−1^, while control wells received an equivalent volume of phosphate-buffered saline (PBS). The 6 U mL^−1^ dose was selected based on prior optimization studies demonstrating at least 90% degradation of AHLs (C6-homoserine lactone to C12-homoserine lactone) and the absence of cytotoxicity in human gingival fibroblasts and human gingival epithelial cells [22,23,25]. Plates were incubated anaerobically at 37 °C for 48 h. Glass coverslips were selected for biofilm cultivation due to their standardized surface properties, which facilitate reproducible biofilm formation and imaging. All procedures after sample collection were performed in an anaerobic chamber (80% N_2_, 10% H_2_, 10% CO_2_) using pre-reduced BHI broth supplemented with 5 μg mL^−1^ hemin and 1 μg mL^−1^ vitamin K_1_ that had been pre-equilibrated under anaerobic conditions for over 24 h prior to use.

### 2.5. Microbiome Analysis

Biofilms were gently washed with PBS to remove loosely adherent cells. DNA was extracted using the MagBeads FastDNA Kit (MP Biomedicals Company, Solon, OH, USA). The standard V3–V4 regions of 16S rRNA were amplified using the barcoded primer pair of 338F (5′-ACTCCTACGGGAGGCAGCA-3′) and 806R (5′-GGACTACHVGGGTWTCTAAT-3′). Samples were sequenced on NovaSeq 6000 (Illumina Company, San Diego, CA, USA). The raw datasets were processed with DADA2 pipeline for filtering and trimming, merging paired reads, and constructing an Amplicon Sequence Variant (ASV) table. Taxonomy assignment of sequences was performed with the assign taxonomy function on a curated train set with species of the SILVA database v138.2 for DADA2. An unrooted maximum likelihood phylogenetic tree was generated using FastTree. For downstream microbiome analysis, singletons in data were filtered out retaining a final count of 3300 ASVs. Details are described in Supplementary Materials.

### 2.6. Biofilm Characterization

The biofilm morphology, biomass, and kinetics were studied using scanning electron microscopy (SEM), crystal violet assay, and confocal laser scanning microscopy (CLSM). For SEM evaluation, the biofilms were fixed in 2.5% glutaraldehyde at 4 °C for 30 min, dehydrated in an ethanol gradient, critical-point dried, and sputter-coated with gold for evaluation using SEM (GeminiSEM 300, ZEISS, Oberkochen, Germany). The crystal violet assay was used to quantify the biomass of the biofilms. The biofilms were fixed with methanol, stained with 1% crystal violet for 15 min, and dissolved in 95% ethanol. Absorbance at 590 nm (OD_590_) was measured to quantify biomass. CLSM was performed using a ZEISS LSM880 microscope (ZEISS, Oberkochen, Germany). Biofilms were stained with SYTO 9 (11 μM) and propidium iodide (66 μM) (LIVE & DEAD Baclight Bacterial Viability Kit, L7012 Molecular Probes, Invitrogen, Waltham, MA, USA).

### 2.7. Statistical Analysis

Biofilm biomass and thickness were compared using Student’s *t*-test (GraphPad Prism 8.0). Alpha diversity metrics were analyzed with the Mann–Whitney U test, and beta diversity (Weighted UniFrac distances) was visualized via a principal coordinate analysis (PCoA). The 16S rDNA sequencing data analyses were performed using the QIIME2 and R packages (V3.3.2). The taxa composition at the phylum, family, and genus level was visualized using ‘QIIME taxa barplot’. Functional predictions were generated using Phylogenetic Investigation of Communities by Reconstruction of Unobserved States (PICRUSt2). Statistical significance was set at *p* < 0.05.

The sample size was calculated using GPower 3.1.9.7. One of the main outcomes measured was biomass using crystal violet staining. The preliminary in vitro data (5 samples per group) showed that Est816 reduced biofilm biomass from 2.57 ± 0.34 (control) to 1.86 ± 0.25 (OD_590_), corresponding to Cohen’s d = 2.37. To conservatively account for clinical variability and potential effect attenuation, we assumed Cohen’s d = 0.8. With α = 0.05 and 80% power, a two-tailed independent *t*-test indicated 25 samples per group were required. To balance statistical rigor with clinical feasibility, we increased the number of participants enrolled by 20%, resulting in a total of 30 participants.

## 3. Results

### 3.1. Diversity Changes

**Figure 1:**
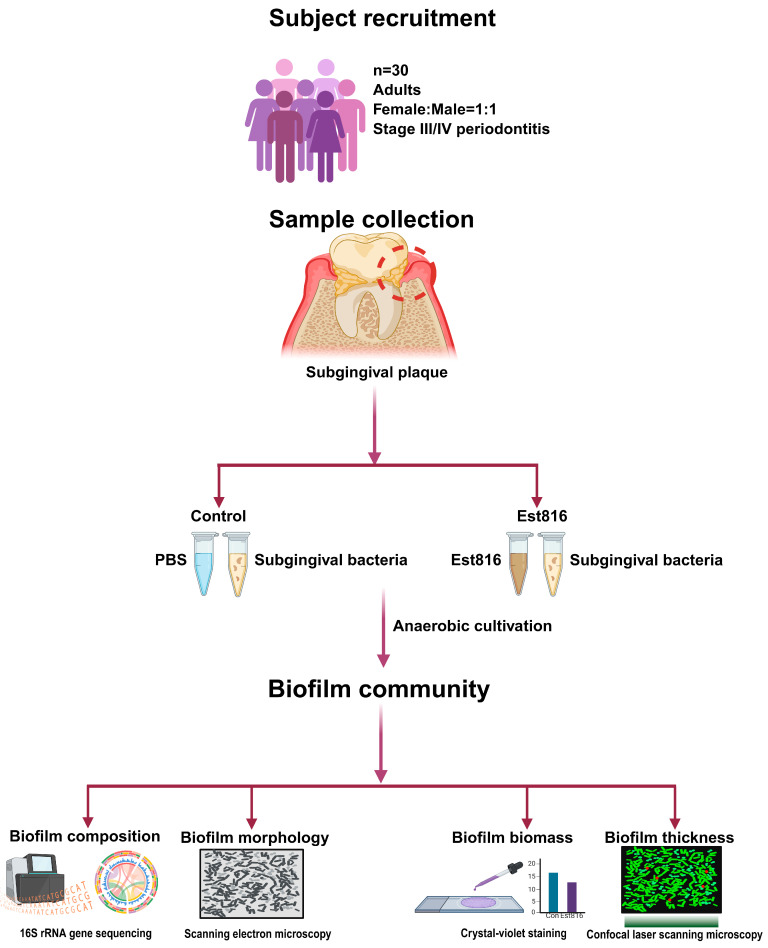
Diagram of subgingival plaque sample collection and laboratory process (n = 30).

**Figure 2:**
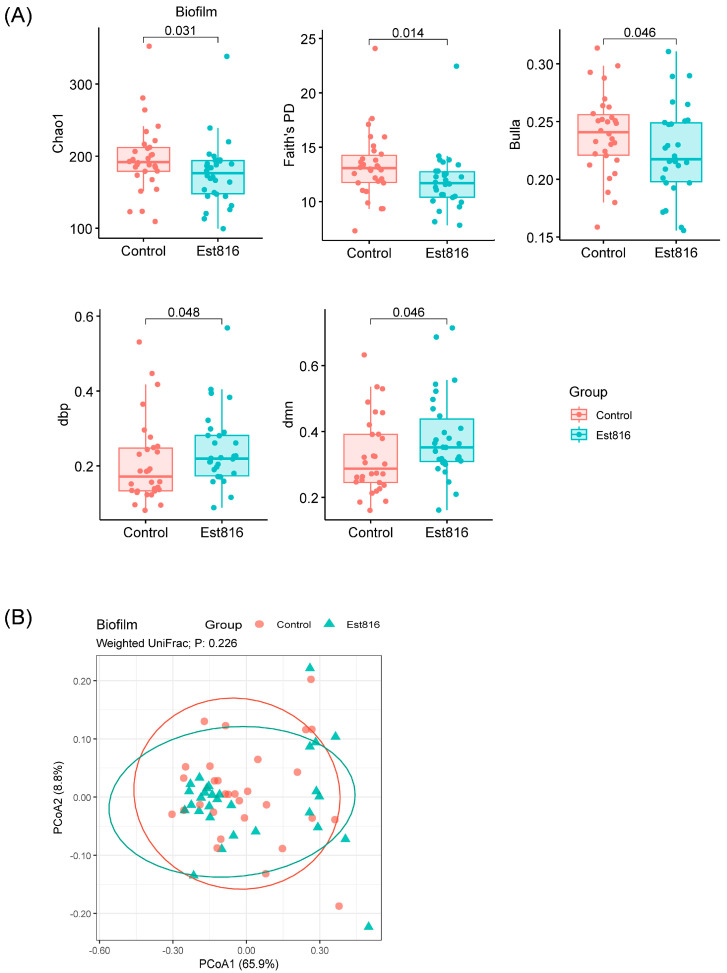
(**A**) Alpha diversity of biofilm samples. The significances between control and Est816 groups were evaluated using Mann–Whitney U Test. Richness index: Chao1; diversity index: Faith’s phylogenic diversity; evenness index: Bulla; dominance indices: Berger–Parker Index (dbp) and McNaughton’s index (dmn). (**B**) PCoA of Weighted UniFrac distances of biofilm samples of treatment groups of control and Est816. PERMANOVA revealed no significant differences. Each group contained 30 samples.

In total, 30 participants were recruited, with a balanced distribution of 15 males and 15 females and a mean age of 46 ± 13 years (Table 1). There was an almost equal distribution of gender and an almost equal distribution of age and types of periodontitis among these 30 participants. Thirty subgingival plaque samples were collected (Figure 1). The microbiome composition of biofilm culture of subgingival plaques was analyzed using 16S rRNA amplicon sequencing. The singleton-removed dataset successfully removed batch effects for downstream analyses (Figure S1). Est816 treatment significantly altered alpha diversity indices in biofilm samples compared to the control group (*p* < 0.05). Specifically, the Chao1 Index, Faith’s PD Index, Bulla’s Index, and dominance metrics (dbp and dmn Index) showed notable changes following treatment (Chao1 Index (176 [147–194] vs. 192 [179–212], *p* = 0.031), Faith’s PD Index (11.7 [10.4–12.8] vs. 13.1 [11.7–14.4], *p* = 0.014), Bulla’s Index (0.22 [0.20–0.25] vs. 0.24 [0.22–0.26], *p* = 0.046), dbp (0.22 [0.17–0.28] vs. 0.17 [0.13–0.25], *p* = 0.048), and dmn (0.35 [0.31–0.45] vs. 0.29 [0.24–0.39], *p* = 0.046)). The observed differences highlighted the impact of Est816 on biofilm community structure, reducing richness and diversity while influencing dominance patterns (Figure 2A). There was no significant variation on Weighted UniFrac distances by the PCoA and PERMANOVA (Figure 2B).

### 3.2. Microbial Shifts

**Figure 3:**
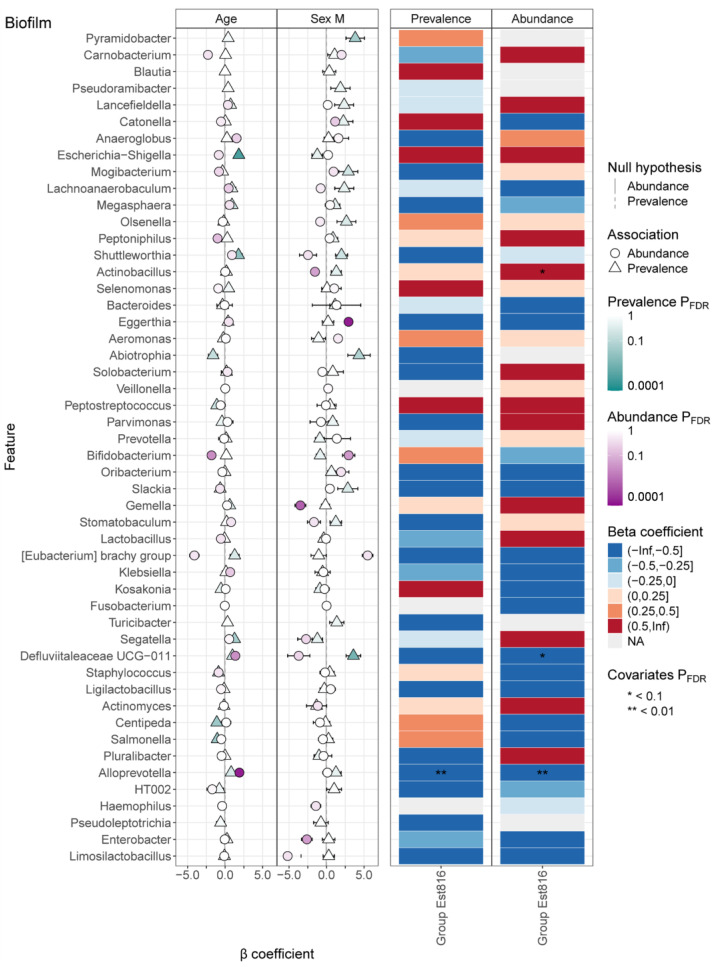
MaAsLin3 selected differentially abundant genera in biofilm samples. The estimated coefficients and their standard errors of selected genera with covariates are represented by points and bars on the left panel. The associations of selected genera with treatment group Est816 relating to control are shown in the right heatmap panels. Both prevalence and abundance were evaluated. Each group contained 30 samples.

Further selection of differentially abundant genera using MaAsLin3, with adjustment for covariates including age and sex, revealed significant associations between microbial abundance and experimental variables (Figure 3). Beta coefficient ranges highlighted distinct directional effects, with strong negative associations (β ≤ −0.5) observed in certain taxa (e.g., *Defluviitaleaceae UCG-011* and *Alloprevotella*, associated with inflammatory modulation), suggesting suppression under Est816 treatment. However, strong positive associations (β ≥ 0.5) were detected in taxa like *Actinobacillus* (associated with biofilm pathogenicity), indicative of enrichment. Covariate adjustment corrected the effects of age and sex on these associations, demonstrating the interplay between host-specific factors and treatment effects. For instance, taxa such as *Eggerthia* and *Gemella* showed sex-dependent abundance shifts, while age influenced trends in *Escherichia-Shigella* and *Centipeda*. Overall, these results demonstrated the taxon-specific responses to Est816 and provided mechanistic insights into its impact on biofilm microbiota composition and stability.

### 3.3. Metabolic Effects

**Figure 4:**
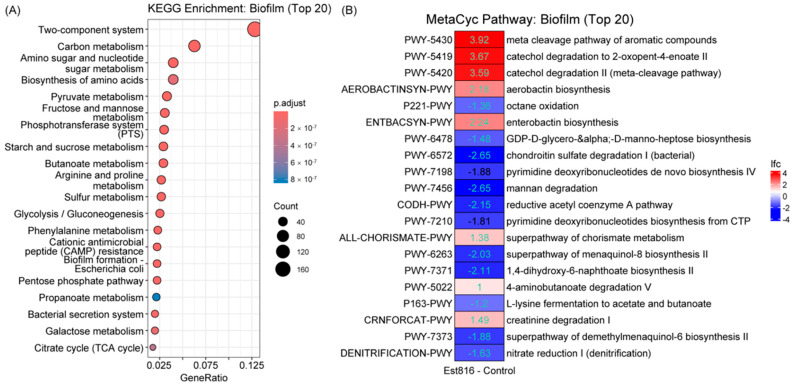
ANCOM-BC2-selected PICRUSt2 prediction. (**A**) KEGG enrichment of significantly differentially abundant KO terms. (**B**) Significantly differentially abundant MetaCyc pathways. ANCOM-BC2 selection criteria: q value (FDR) < 0.2 and passed sensitivity analysis for the pseudo-count addition. lfc: natural logarithm of the fold change. Aquamarine: passed sensitivity analysis. Each group contained 30 samples.

The functional enrichment analysis of biofilm-associated metabolic pathways revealed distinct alterations induced by Est816 treatment compared to the control. The KEGG pathway analysis (Figure 4A) highlighted enrichment in key metabolic processes, including carbon metabolism, amino acid biosynthesis, and two-component systems (TCSs), which are critical for bacterial signal transduction and environmental adaptation. Notably, butanoate metabolism was significantly enriched. Butyrate, a key product of this pathway, is both a virulence factor and energy source for periodontal pathogens like *Porphyromonas gingivalis* and *Fusobacterium nucleatum*, where it promotes inflammation and sustains bacterial survival in periodontal biofilms. Est816 further disrupted bacterial TCSs, impairing quorum-sensing signal detection and downstream responses. Pathways linked to biofilm pathogenicity, such as cationic antimicrobial peptide (CAMP) resistance and biofilm formation, were also enriched, suggesting that Est816 may modulate microbial defense mechanisms and structural stability. The MetaCyc pathway analysis (Figure 4B) further identified differential activity in specialized metabolic networks. Notably, biosynthetic pathways were upregulated, including aerobactin biosynthesis and enterobactin biosynthesis, both associated with iron acquisition and bacterial virulence. Conversely, catabolic pathways such as mannan degradation and nitrate reduction I (denitrification) were suppressed, indicating reduced carbohydrate utilization and anaerobic respiration under Est816 treatment. Overall, these findings demonstrated that Est816 modulated biofilm metabolic activity, favoring pathways linked to virulence factor production while suppressing those involved in nutrient acquisition, stress adaptation, and structural maintenance.

### 3.4. Antibiofilm Activity

**Figure 5:**
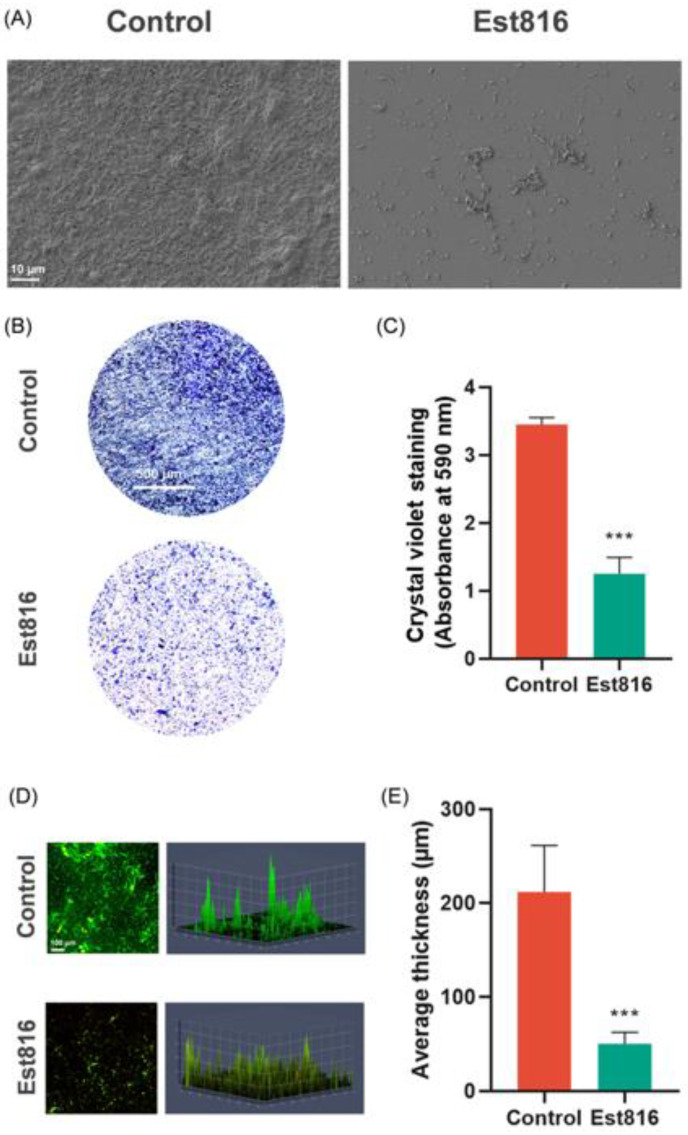
Effect of Est816 on biofilm morphology, biomass, and kinetics. (**A**) The SEM images of biofilm model from participant no. 5 (P5; female, 36 years old, periodontitis Stage III) with original magnification of 1000. (**B**) Photographic images of crystal violet staining of biofilm models from participant no. 5 with original magnification of 5. (**C**) Spectrophotometer quantitative analysis of crystal violet staining of biofilm quantity (compared with control group, *** *p* < 0.001, error bars in each panel represent the SD of 30 samples). (**D**) Confocal microscopy visualization of biofilm growth from clinical subgingival plaque (original magnification of 10). (**E**) Quantitative analysis biofilm thickness by CLSM (compared with control group, *** *p* < 0.001, error bars in each panel represent the SD of 30 samples). Biofilm biomass and thickness were compared using Student’s *t*-test (GraphPad Prism 8.0).

SEM imaging revealed that untreated biofilms exhibited dense, well-structured colonies. In contrast, biofilms exposed to Est816 showed disrupted architecture, with reduced biomass and a fragmented matrix (Figure 5A). The biomass of biofilms was visualized and quantitatively assessed using crystal violet staining. Est816 resulted in a significant reduction by 64% in biofilm biomass compared to the control group (OD_590_: 1.25 ± 0.24 vs. 3.45 ± 0.10; *p* < 0.001) (Figure 5B,C). CLSM imaging showed that untreated biofilms exhibited a thick, continuous structure with uniform distribution of bacterial cells. In contrast, biofilms treated with Est816 showed a significant reduction by 76% in thickness (50 ± 12 µm vs. 212 ± 50 µm, *p* < 0.001), along with a more dispersed and irregular distribution of bacterial clusters (Figure 5D,E). These findings indicated that Est816 inhibited these bacteria from forming dense biofilms.

## 4. Discussion

This study demonstrated that AHL-lactonase Est816 disrupts key QS pathways, inducing profound structural, compositional, and functional remodeling of polymicrobial subgingival-plaque-derived biofilm. Muras et al. reported shifts in microbial populations following AHL-lactonase Aii20J treatment, using saliva and subgingival samples [13,15]. Notably, changes primarily were observed with *Streptococcus*, dominating 99.74% of the saliva-derived communities, while subgingival biofilms exhibited 45–63% *Streptococcus* proportions. Similarly, Sikdar et al. demonstrated that AHL-lactonases (SsoPox and GcL) led to an increased abundance of commensals and early colonizers, e.g., *Lactobacillales*, *Streptococcus*, and *Actinomyces*, while reducing the prevalence of Gram-negative pathogens [12]. Our findings indicated that Est816 significantly altered alpha diversity metrics, while the beta-diversity analysis (PCoA) revealed no significant separation between groups. Est816 induced taxon-specific shifts in microbial abundance, suggesting the selective modulation of biofilm composition rather than wholesale restructuring. It also extended these observations by revealing genus-specific dynamics modulated by Est816, including the suppression of inflammation-associated taxa (e.g., *Defluviitaleaceae UCG-011*, and *Alloprevotella*). *Porphyromonas gingivalis*, a keystone pathogen in periodontitis, plays a critical role in driving community shifts and inflammatory responses in subgingival plaque. Our MaAsLin3 analysis (Figures S2–S5) revealed that Est816 treatment significantly reduced the abundance of *Bacteroidota* at the phylum level. This finding aligned with prior studies demonstrating that *Porphyromonas gingivalis* relies heavily on QS for biofilm maturation. These changes may reflect ecological rebalance in biofilms, where quorum quenching disrupts cooperative behaviors and pathogen dominance, creating an environment for opportunistic taxa. In our previous study, Est816’s effects on *Aggregatibacter actinomycetemcomitans* in single-species biofilms differed from those observed here in polymicrobial communities, likely due to interspecies interactions (e.g., metabolic cross-feeding or QS interference) that modulate pathogen responsiveness. This underscores the importance of clinically relevant biofilm models [25]. Importantly, the sex- and age-dependent microbial shifts demonstrated the necessity of accounting for host covariates in quorum-quenching interventions, as demographic factors may influence baseline microbiota composition and treatment responsiveness. For instance, the enrichment of *Eggerthia* in females and *Escherichia-Shigella* in older participants may reflect hormonal or immunosenescence effects on immunity [26,27,28]. Covariate adjustments for age and sex revealed trends in taxon-specific responses, though the sample size limits robust conclusions about three-way interactions. Larger cohorts will be needed to validate these associations.

The functional prediction analysis using PICRUSt2 revealed that Est816-treated microbial communities were significantly enriched in metabolic pathways related to amino acid biosynthesis, carbohydrate metabolism, and cofactor pathways. This shift in metabolic functions suggested that Est816 not only altered the taxonomic composition of the subgingival microbiota but also induced a functional reprogramming of its metabolic landscape. Butyrate produced by pathogens like *Porphyromonas gingivalis* activates host pro-inflammatory pathways (e.g., NF-κB) and inhibits fibroblast repair functions [27]. Est816-treated biofilms exhibited reduced activity in butanoate metabolism, a pathway linked to virulence in periodontal pathogens like *Porphyromonas gingivalis*. While butyrate is known to promote host inflammation and bacterial survival in biofilms, our functional data do not establish a direct causal relationship between this metabolic shift and clinical outcomes. Further studies integrating metabolomics and host-response assays are needed to elucidate whether butanoate suppression contributes to Est816’s antibiofilm effects. The concurrent enrichment of stress-response pathways (e.g., aromatic compound degradation) implied compensatory microbial adaptations to quorum-quenching-induced disruption, while the downregulation of nucleotide biosynthesis and anaerobic respiration pathways (e.g., nitrate reduction) pointed to impaired metabolic resilience [28,29]. These shifts aligned with the reduced alpha diversity and altered dominance patterns observed in Est816-treated biofilms, indicating that Est816 destabilized metabolic interdependencies essential for community stability. The upregulation of iron-scavenging pathways (e.g., aerobactin/enterobactin biosynthesis) may reflect resource competition under treatment stress, further destabilizing pathogen networks reliant on host-derived nutrients [30,31].

Moreover, Est816 caused profound biofilm disintegration, reducing biomass by 64% and thickness by 76%. This disruption may increase biofilm permeability, enhancing susceptibility to host immune effectors and adjunct therapies. Notably, the fragmented architecture observed aligned with suppressed TCSs pathways, which coordinate biofilm maturation and extracellular matrix production. By impairing TCS-mediated signaling, Est816 may prevent bacterial populations, creating a vulnerable, disorganized biofilm state [32,33,34,35]. Such a property is particularly critical in periodontal disease, where biofilm resilience plays a central role in chronic infections and treatment resistance [36]. Thus, this mechanism could synergize with mechanical debridement, as disrupted biofilms may be more physically removable from subgingival pockets. While glass coverslips provide a standardized surface for biofilm growth, they lack tooth-like physicochemical properties (e.g., roughness and hydrophobicity). Future studies could use hydroxyapatite-coated substrates to better mimic subgingival conditions. Although our study demonstrated Est816’s antibiofilm effects, the absence of a heat-inactivated or mutated enzyme control means we cannot rule out nonspecific protein effects. Future work could include these controls to confirm the specificity of Est816’s quorum-quenching activity. The sample size of our study may limit its generalizability to periodontitis populations. Future studies with larger, stratified cohorts are needed to validate these findings across more diverse populations.

Est816 altered the biofilm’s taxonomic and functional profile, demonstrating antibiofilm activity. Meanwhile, several critical considerations emerged. First, the observed enrichment of *Actinobacillus* following treatment suggested potential risks of unintended microbial shifts, where the suppression of dominant pathogens may create opportunities for secondary colonizers. Est816’s specificity for AHLs suggests minimal disruption to commensal biofilms in healthy sites, as Gram-positive commensals (e.g., *Streptococcus sanguinis*) lack AHL-based QS. However, in vivo studies are needed to confirm ecological safety. Future strategies could combine Est816 with targeted antimicrobials or probiotics to maintain ecological balance, consider dosage optimization to balance QS disruption with ecological stability, and develop targeted delivery systems to minimize the exposure of commensal microbiota. Future studies could include multi-site sampling to assess broader ecological impacts. Second, bacterial stress-response upregulation indicated possible metabolic adaptations that might affect long-term efficacy, warranting further investigation through multi-omics approaches. Est816 disrupts communication without lethal pressure, potentially reducing resistance risk. However, compensatory mutations in QS receptors (e.g., *luxR* homologs) or biofilm matrix overproduction could occur. Combining Est816 with mechanical debridement or probiotics may mitigate this.

While in vitro models demonstrated Est816’s antibiofilm capacity, its clinical application requires overcoming several translational challenges. The artificial nature of our experimental system warrants consideration. While BHI broth supports the growth of many periodontal pathogens, it may not equally sustain all species present in native subgingival plaque, particularly fastidious organisms like spirochetes. Also, the periodontal pocket harbors a complex polymicrobial consortium producing diverse proteases whose potential effects on Est816 stability require systematic investigation. Future studies should evaluate enzyme stability in this proteolytic environment. Additionally, we need to develop drug delivery systems capable of maintaining long-term therapeutic concentrations through sustained release mechanisms. While recognizing that our polymicrobial subgingival-plaque-derived biofilm model undergoes inevitable microbial composition changes during in vitro cultivation, this pre-clinical study provides essential proof-of-concept data on quorum quenching in clinically relevant microbial communities. The observed biofilm modulation effects of Est816, albeit in an artificial system, establish important groundwork for future animal studies and clinical translation.

## 5. Conclusions

In conclusion, this study elucidates the role of the quorum-quenching enzyme AHL-lactonase Est816 in modulating the composition and dynamics of polymicrobial biofilm derived from subgingival plaque samples. Est816 disrupted structural integrity and metabolic coordination in patient-derived periodontal biofilms in vitro, demonstrating potential as a biofilm-targeting agent.

## Figures and Tables

**Table 1:** Age range and sex of the 30 participants by stage of periodontitis.

Stage	Number of Participants	Age	Sex
Stage III	19	29–51	10 × Male
			9 × Female
Stage IV	11	54–75	5 × Male
			6 × Female

Periodontitis status was assessed according to the proceedings from The American Academy of Periodontology and the European Federation of Periodontology Classification of Periodontal and Peri-implant Diseases 2017.

## Data Availability

The data presented in this study are available upon request from the corresponding author.

## Supplementary Materials

The following supporting information can be downloaded at https://www.mdpi.com/article/10.3390/dj13080372/s1, Supplementary Materials; Figure S1 Batch effect removal using ConQuR; data of biofilm samples were processed separately; Figure S2 Average relative abundance at levels of phylum, family, and genus; Figure S3 Heat map of standardized clr (clr-z) of phyla; Figure S4 MaAsLin3 selected differentially abundant families in biofilm samples; Figure S5 Differentially abundant families and genera in biofilm samples.
