# Supplementary material for: Quorum-Quenching AHL-Lactonase Est816 Inhibits Polymicrobial Subgingival-Plaque-Derived Biofilm Formation
Source: Dent J (Basel). 2025 Aug 15;13(8):372. doi: 10.3390/dj13080372 (PMC12385154; doi:10.3390/dj13080372)
Supplement: Supplementary file 1 [file dentistry-13-00372-s001.zip › dentistry-3706902-supplementary.pdf]

Supplementary Materials

Materials and Methods

Data processing and microbiome analysis

Batch effects were removed using ConQuR v.2.0 (https://www.nature.com/articles/s41467-022-33071-9) package on datasets of biofilm samples separately with covariate adjustment of sex and age of sampling patients. Alpha-diversity indices were calculated using addAlph function of mia v.1.14.0 package. The overall microbiome structure was examined on Weighted UniFrac distances by principal coordinate analysis (PCoA) using vegdist function and Permutational Multivariate Analysis of Variance (PERMANOVA) using adonis2 function of vegan v2.6-4.

Differentially abundant taxa between two groups (Control and Est816) were selected using MaAsLin3 (https://www.biorxiv.org/content/10.1101/2024.12.13.628459v1) relative abundance data, as normalization = 'TSS', transform = 'LOG', min_prevalence = 0.05, max_significance = 0.1 with covariate adjustment of sex and age of sampling patients. Unstratified microbial functional prediction was performed using PICRUSt2 full pipeline on Galaxy platform (usegalaxy.eu) and the predicted KEGG orthologs (KOs) and MetaCyc pathways (PWs) were further assessed using ANCOM-BC2 ($q < 0.2$) with covariate adjustment of sex and age. Significant KOs were further enriched with KEGG pathway using clusterProfiler v.4.13.0.

Figures

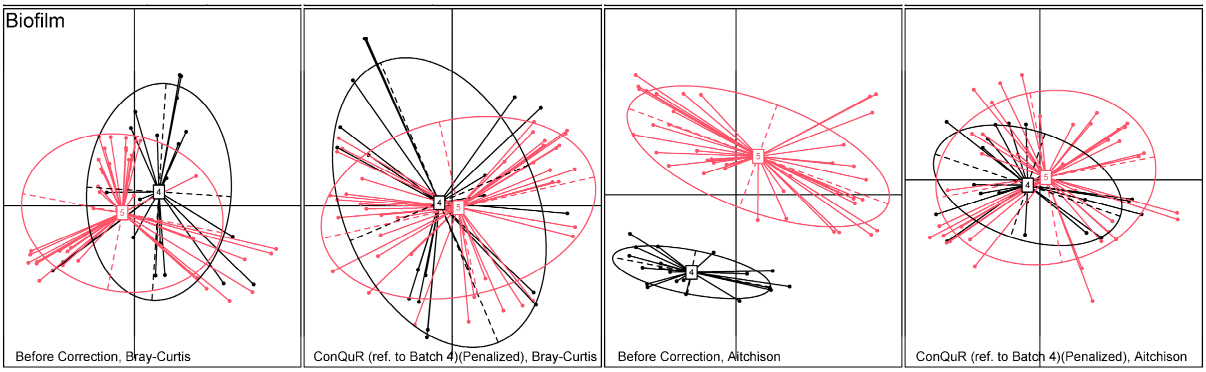

Figure S1 Batch effect removal using ConQuR. Data of biofilm samples were processed separately. The performance of removal was presented as PCoA of Bray-Curtis and Aitchison distances respectively. The best performance for biofilm samples was achieved using penalized ConQuR using logistic_lasso.

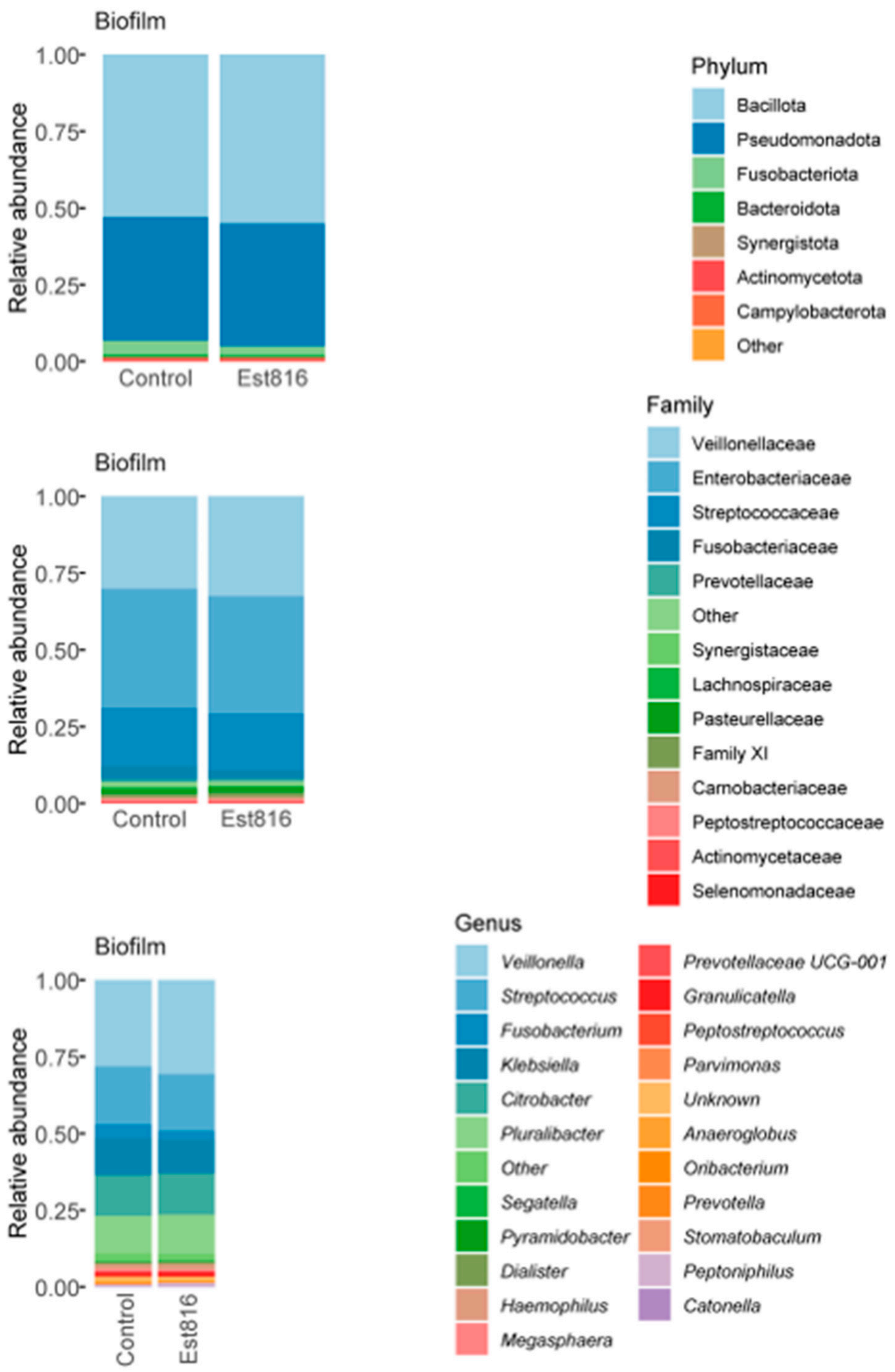

Figure S2 Average relative abundance at levels of phylum, family and genus. For biofilm samples, relative abundance was averaged by treatment groups of Control and Est816.

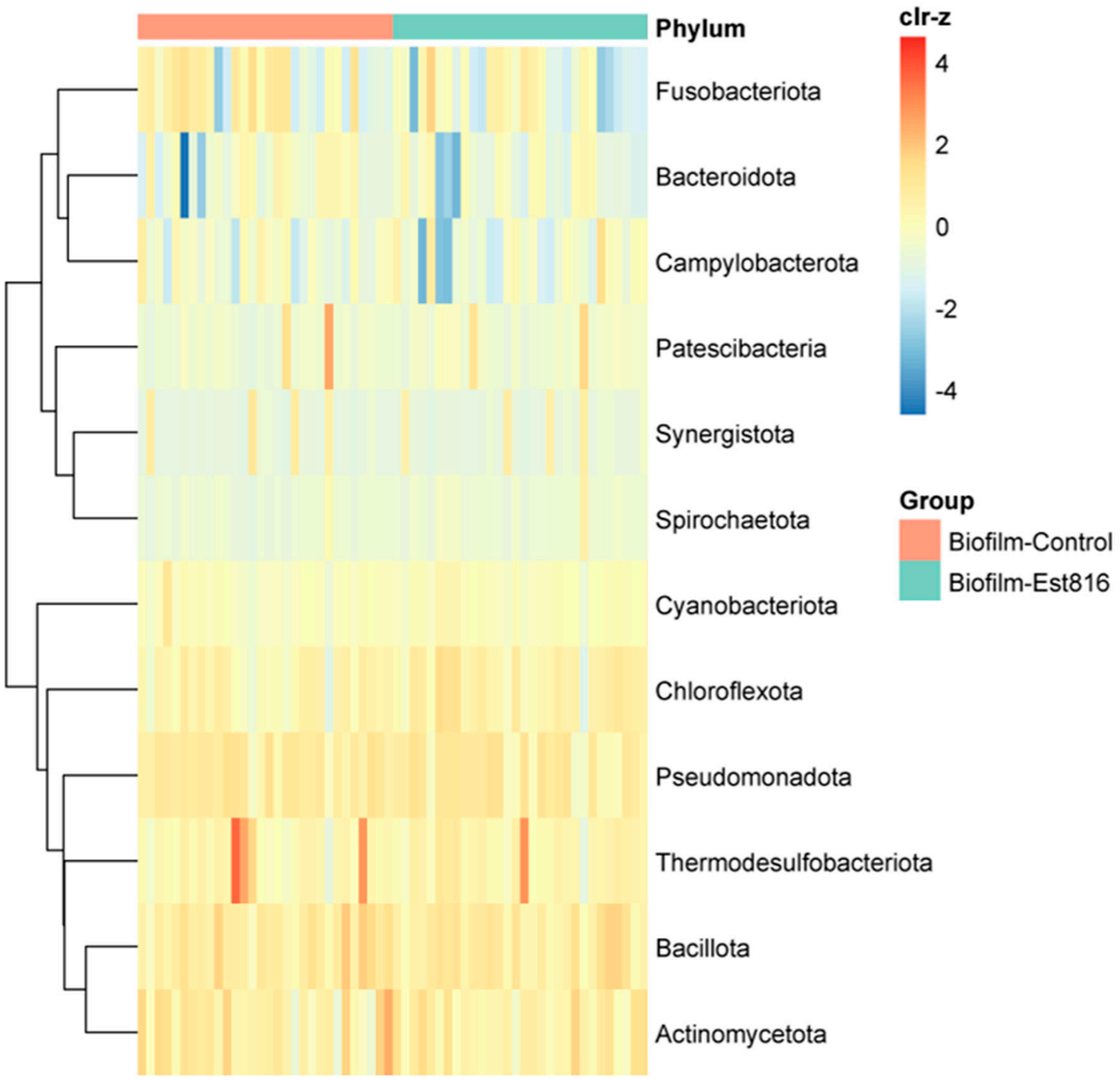

Figure S3 Heat map of standardized clr (clr-z) of phyla.

Samples are annotated by groups of Biofilm-Control, Biofilm-Est816.

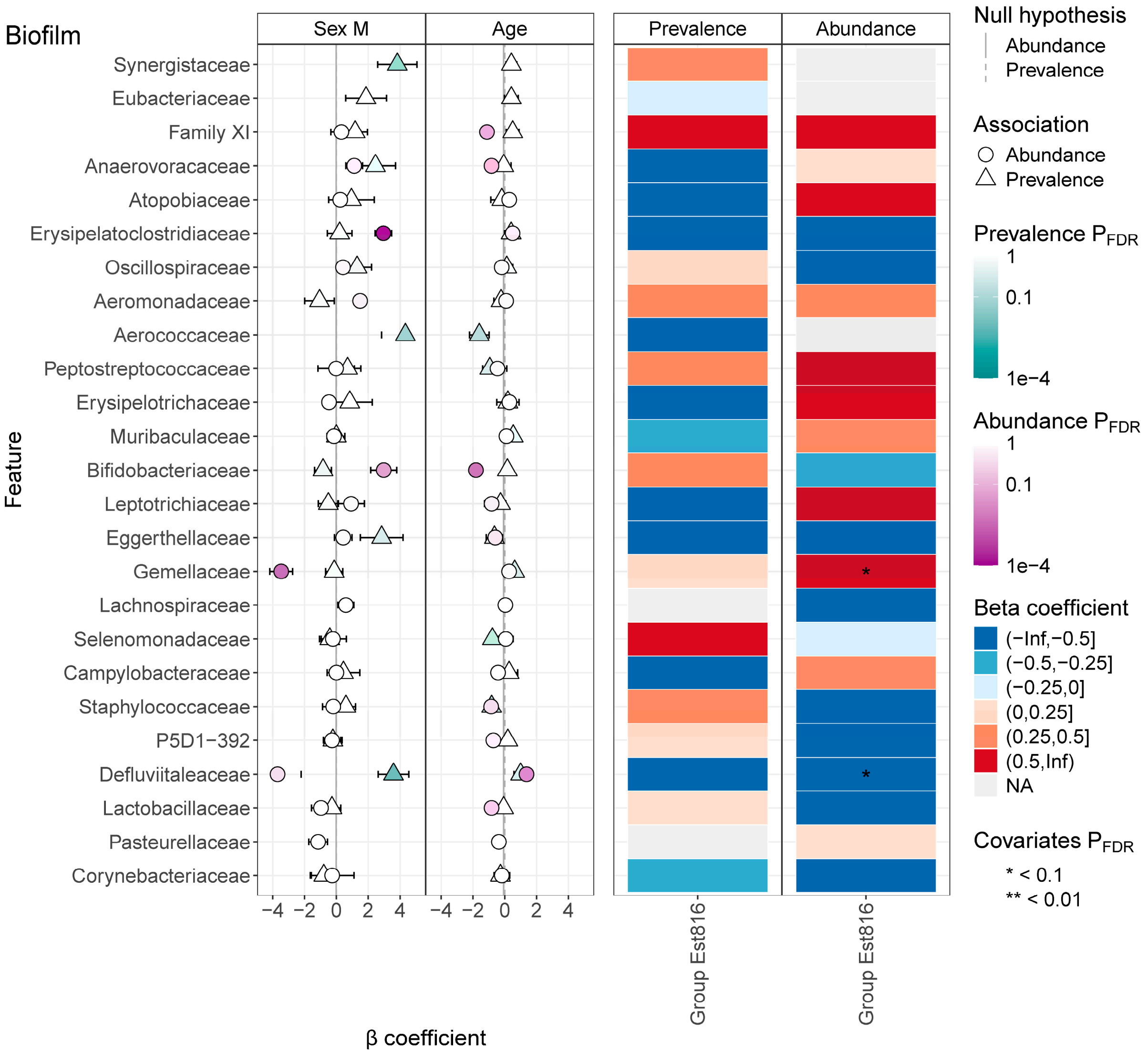

Figure S4 MaAsLin3 selected differentially abundant families in biofilm samples. The estimated coefficients and their standard errors of selected genera with covariates were represented by points and bars on the left panel. The associations of selected genera with treatment group Est816 relating to Control were shown in the right heatmap panels. Both prevalence and abundance were evaluated.

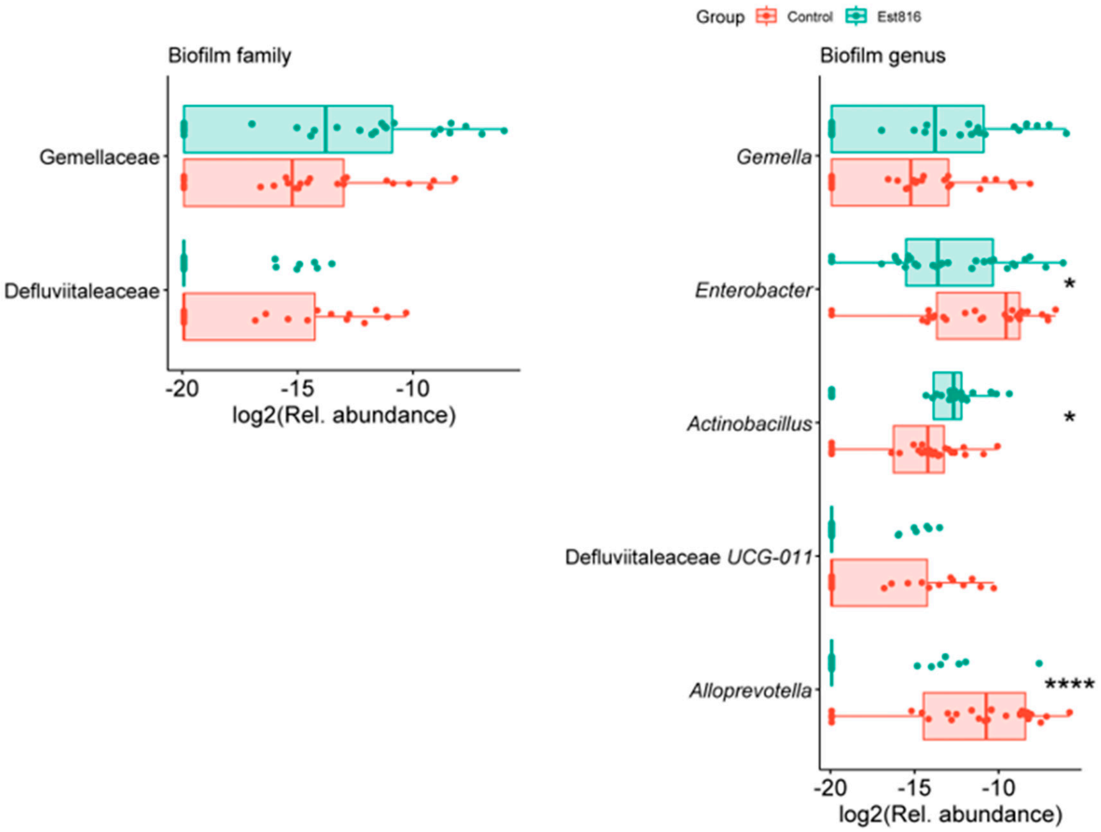

Figure S5 Differentially abundant families and genera in biofilm samples. MaAsLin3 analysis identified taxa significantly associated with the treatment group Est816 compared to Control. Points represented log2-transformed relative abundance differences (Est816 vs. control), with statistical significance (**p* < 0.05, ***p* < 0.01, ***p* < 0.001). Only taxa meeting significance thresholds (after FDR correction) were shown.
